# Reviving the Standstill Heart: Multimodal Management of Refractory Cardiac Arrest due to Massive Pulmonary Embolism

**DOI:** 10.1155/crcc/6192253

**Published:** 2026-08-27

**Authors:** Jeffrey Valencia Uribe, Mariam Attia, Alfredo Perez Tagle Tejeda, Kyle Seifert, Ramon G. Valentin, Daniel Mayer

**Affiliations:** ^1^ Internal Medicine, Memorial Hospital West, Pembroke Pines, Florida, USA; ^2^ Emergency Medicine, Memorial Hospital West, Pembroke Pines, Florida, USA; ^3^ Critical Care Medicine, Memorial Hospital West, Pembroke Pines, Florida, USA

## Abstract

**Introduction:**

Massive pulmonary embolism (PE) is a life‐threatening condition frequently associated with right ventricular (RV) strain, hypotension, and cardiac arrest. Recommended management includes IV alteplase for high‐risk PE. However, evidence guiding higher thrombolytic doses during cardiac arrest remains limited and is rarely reported. We present a case of massive PE requiring an atypically high cumulative tPA dose administered during CPR, resulting in return of spontaneous circulation (ROSC).

**Case Description:**

A 38‐year‐old female with prior DVT and PE presented with syncope and left lower extremity edema. Imaging confirmed bilateral PE and DVT. She was found to be hemodynamically unstable and was treated with 100 mg IV tPA. This resulted in transient improvement, but she subsequently suffered a prolonged PEA arrest. During ACLS, there was confirmation of cardiac standstill on POCUS. A second bolus of 50 mg tPA was administered 29 min into the code, which led to sustained ROSC within 2 min. Despite maximal vasopressor and inotropic support, persistent shock and hypoxemia necessitated pulmonary angiography and attempted thrombectomy, which was aborted due to recurrent arrest. Catheter‐directed thrombolysis with bilateral EKOS catheters was initiated, and by ICU Day 3, echocardiography revealed normalized RV function.

**Discussion:**

Massive PE causes acute RV failure and impaired pulmonary perfusion, which may limit thrombolytic delivery. Repeat tPA dosing during CPR may enhance clot penetration and improve outcomes. When systemic thrombolysis is insufficient, catheter‐directed therapies can provide additional hemodynamic benefit. Adjunctive strategies including ventilatory optimization, pulmonary vasodilation, and neuroprotection were critical for the patient to achieving full recovery.

**Conclusion:**

Aggressive, multidisciplinary management, including repeat thrombolysis during arrest, can overcome traditionally poor prognostic indicators in massive PE and result in favorable neurologic outcomes.

## 1. Introduction

Massive pulmonary embolism (PE) is a life‐threatening condition that can rapidly progress to obstructive shock, cardiac arrest, and death. Massive PE causes a sudden elevation in pulmonary vascular resistance (PVR) due to mechanical obstruction, leading to right ventricular (RV) strain, dilation, and eventual failure. As RV pressures rise, the interventricular septum bows toward the left ventricle (LV), hindering its filling. This, along with elevated pericardial pressure and right atrial congestion, reduces LV output and results in systemic hypotension characterized by obstructive and cardiogenic shock. Additionally, increased RV wall stress and transmural pressure impair coronary perfusion, contributing to myocardial ischemia.

Hypoxia results from regional bronchoconstriction, reduced cardiac output, and a ventilation‐perfusion mismatch, which collectively increase dead space ventilation. Given this pathophysiologic cascade, clinical evidence of RV dysfunction and hemodynamic instability is associated with a markedly elevated risk of early mortality.

The ACCP recommends primary thrombolysis with IV alteplase (tPA) 100 mg over 2 h for massive PE [1]. Higher cumulative thrombolytic doses are rarely documented. This case describes a patient with massive PE who received the standard loading dose and an additional infusion dose of alteplase. The patient subsequently received a second bolus dose during cardiac arrest, resulting in return of spontaneous circulation (ROSC) and ultimately full recovery.

## 2. Case Description

**Figure 1:**
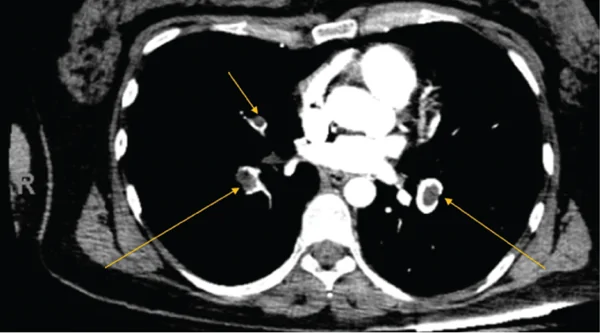
CTA chest pulmonary embolism with IV contrast revealed bilateral PE (yellow arrows).

**Figure 2:**
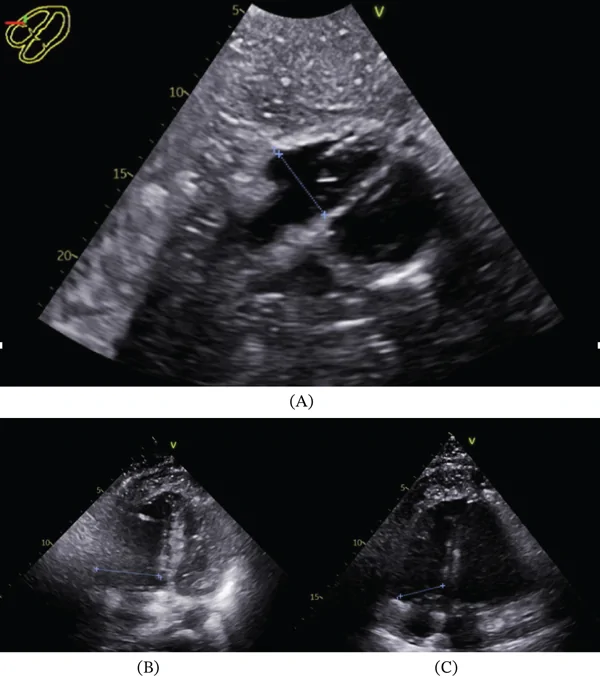
(A) Initial subcostal echocardiogram demonstrating a moderately dilated RV with severely reduced systolic function. (B) Apical four‐chamber view showing an RV base diameter of 4.29 cm (normal: 3.5–4 cm) and tricuspid annular plane systolic excursion (TAPSE) of 11.1 mm (abnormal < 16 mm). (C) Repeat apical view 3 days posttreatment with RV base reduced to 3.08 cm and TAPSE improved to 19.82 mm, consistent with recovery of RV function.

A 38‐year‐old woman with a history of unprovoked deep vein thrombosis (DVT) and PE in 2015—previously treated with anticoagulation for 6 months before self discontinuation and subsequent loss to outpatient follow‐up for thrombophilia evaluation—presented to the emergency department after a syncopal episode associated with pain and edema of the left lower extremity (LLE). The patient was found to be tachycardic, hypotensive, and tachypneic, but saturating well. On physical exam, she appeared acutely ill and in respiratory distress. Initial labs revealed lactic acidosis of 9.7 mmol/L and a troponin of 0.037 ng/mL. Due to concerns for PE, a CT aortoiliac runoff was performed, revealing LLE DVT, and computed tomography angiography (CTA) of the chest revealed extensive bilateral PE (Figure 1). An echocardiogram revealed a severely dilated RV and decreased right ventricular systolic function (RVSF) with McConnell′s sign (Figure 2).

She received 100 mg IV tPA with transient improvement, but then deteriorated with a prolonged pulseless electrical activity (PEA) arrest approximately 1 h after completion of the infusion. She was intubated, received multiple rounds of ACLS, and cardiac standstill was confirmed on POCUS. There were transient episodes of ROSC but quick reversion to PEA arrest. At 29 min into the code, a second 50 mg IV tPA was administered, with eventual ROSC achieved. The total duration of cardiopulmonary resuscitation (CPR) was 41 min. Due to obstructive and cardiogenic shock, she required high doses of epinephrine, norepinephrine (NE), and vasopressin (AVP) together with milrinone for inotropic RV support. Hypoxemic respiratory failure was managed with low positive end‐expiratory pressure (PEEP) and low tidal volume to avoid increased intrathoracic pressure. Furthermore, nebulized epoprostenol and inhaled nitric oxide (NO) were administered to lower PVR.

**Figure 3:**
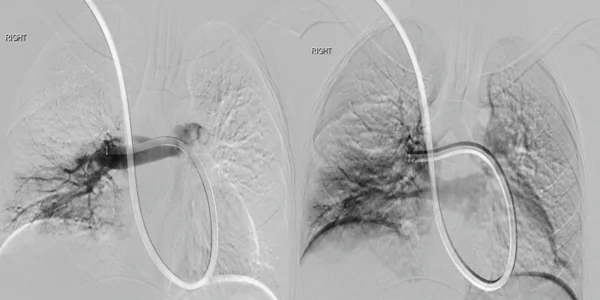
Pulmonary angiogram revealed bilateral filling defects in the pulmonary vasculature.

Despite ongoing treatment, due to worsening shock and hypoxemia, the extracorporeal membrane oxygenation (ECMO) team was contacted for evaluation; however, mobilization of personnel and resources was estimated to require 3–4 h before cannulation could be performed, which would have delayed definitive management with thrombectomy. In addition, the patient was considered to be at high risk for hemorrhage after receiving 150 mg of intravenous tPA. Interventional radiology therefore proceeded with pulmonary angiography (Figure 3), 90 min after ROSC was achieved. However, the procedure was aborted due to a second PEA arrest, from which ROSC was achieved after one round of CPR. Interventional radiology subsequently proceeded with catheter‐directed thrombolysis using bilateral EkoSonic Endovascular System (EKOS) catheters, with tPA infused at 1 mg/h through each catheter for 14 h. An unfractionated heparin infusion was also initiated during this period.

Over the following 24 h, the patient developed a femoral arterial line hematoma, associated with a hemoglobin decrease from her baseline of 9.0 to 6.7 g/dL, requiring transfusion of one unit of packed red blood cells during the hospitalization. There was no evidence of intracranial hemorrhage, large gastrointestinal bleeding, hemopericardium, or massive hemoptysis. The heparin infusion was held in the setting of the hemoglobin decrease. She also developed multiorgan failure with renal failure requiring continuous renal replacement therapy and severe ischemic hepatopathy. On hospital Day 3, repeat echocardiography in the intensive care unit (ICU) demonstrated normalization of RV function, and the patient was completely weaned off vasopressor support by hospital Day 4. Anticoagulation was resumed on hospital Day 5 once aPTT normalized, with argatroban because of concern for heparin induced thrombocytopenia (HIT), given a greater than 50% decrease in platelet count from 226 × 10^9^/L on admission to 51 × 10^9^/L by hospital Day 5. Argatroban therapy was monitored using the aPTT, with a target of 1.5–3 times the baseline value, not exceeding 100 s. HIT was ultimately ruled out, and she was subsequently transitioned to apixaban.

During her time in the ICU neuroprotective measures were employed, including normothermia, normoglycemia, normocapnia, and elevating the head of the bed to more than 30° to support cerebral perfusion and recovery. By hospital Day 6, the patient was liberated from mechanical ventilation to high flow nasal cannula and was neurologically intact. She was downgraded from the ICU on hospital Day 9 and received aggressive physical therapy, progressing to ambulation with minimal assistance. She was discharged on hospital Day 16 with a cerebral performance category score of 1.

## 3. Discussion

### 3.1. Antithrombotic and Pharmacomechanical Therapy

The PEAPETT study showed that rapid administration of 50 mg tPA during CPR in patients with PEA from massive PE resulted in high ROSC rates, reduced pulmonary pressures, and no recurrence of PE or major bleeding during follow‐up [2]. In PE‐related cardiac arrest, severe RV failure and low‐flow physiology may limit pulmonary perfusion and thrombolytic delivery to the obstructing clot. Repeat alteplase administration during high‐quality CPR may improve thrombolytic exposure during a period of partially restored circulation, potentially reducing clot burden enough to restore RV output and pulmonary blood flow.

In our patient, ROSC was achieved after repeating systemic alteplase; however, she remained in persistent obstructive shock and severe hypoxemia, suggesting an incomplete clinical response to systemic thrombolysis. In accordance with guideline‐supported escalation strategies for massive PE with refractory shock after systemic thrombolysis, catheter‐based intervention was pursued as salvage therapy [1, 3–5]. Aspiration thrombectomy achieved limited clot removal, possibly due to a large or organized thrombus burden. During the procedure, recurrent PEA arrest may have resulted from distal embolization, an abrupt worsening of RV afterload, or procedure‐related pulmonary vascular irritation, all of which can precipitate acute RV failure.

Because mechanical clot extraction was incomplete, bilateral EKOS catheters were placed for ultrasound‐assisted catheter‐directed thrombolysis. Ultrasound‐assisted thrombolysis may enhance fibrin separation and local alteplase penetration while allowing lower dose regional therapy. Prior studies have shown improvement in RV/LV ratio, pulmonary artery pressures, and thrombus burden with low rates of intracranial hemorrhage, although major bleeding remains an important risk, particularly after preceding systemic thrombolysis [6–9]. In our case, the cumulative alteplase exposure was high, consisting of 150 mg systemic tPA followed by 28 mg catheter‐directed alteplase through bilateral EKOS catheters. This may have contributed to the patient′s femoral arterial line hematoma and a decrease hemoglobin, highlighting the need to balance rescue reperfusion against hemorrhagic risk in refractory massive PE.

This case highlights the complexity of managing massive PE with cardiac arrest, persistent shock after systemic thrombolysis, and incomplete mechanical thrombectomy. It adds to limited case‐based evidence suggesting that repeat thrombolysis and sequential catheter‐directed therapy may be considered in selected patients when the risk of death from persistent obstructive shock outweighs the bleeding risk [10]. However, standardized dosing strategies after initial systemic thrombolysis remain undefined, and treatment should be individualized based on hemodynamic response, imaging findings, bleeding risk, and institutional expertise.

## 4. Management of RV Dysfunction in Massive PE

### 4.1. Clinical Significance and Therapeutic Objectives

RV dysfunction is a key driver of morbidity and mortality in the setting of massive PE. Management in the ICU is particularly challenging and lacks universally accepted guidelines. The central therapeutic objectives involve optimizing RV preload and contractility, maintaining systemic arterial pressure to ensure coronary perfusion, and reducing RV afterload [11]. These strategies are essential to prevent pulmonary hypertensive crises, acute cor pulmonale, and progression to biventricular failure and cardiovascular collapse.

### 4.2. Hemodynamic Support With Vasopressors

In our case, careful hemodynamic support was prioritized to preserve right coronary artery perfusion, which is especially susceptible to compromise when PVR approaches or exceeds systemic vascular resistance (SVR) [11]. NE was selected as the primary vasopressor. At low doses (< 0.5 *μ*g/kg/min), NE effectively increased SVR without significantly raising PVR [11]. Its *β*_1_‐adrenergic activity further contributed to improved cardiac output and RV‐arterial coupling. AVP was used as a noncatecholamine adjunct. At low doses, AVP acts on V1 receptors to induce systemic vasoconstriction while simultaneously promoting pulmonary vasodilation via NO‐mediated mechanisms [11]. This dual effect reduced the PVR/SVR ratio and improved RV perfusion, without the tachyarrhythmias often seen with catecholamines. High doses of AVP were avoided to prevent potential coronary vasoconstriction and myocardial ischemia, particularly in the context of cardiogenic shock.

### 4.3. Inotropic Support for RV Contractility

To enhance RV contractility, we employed epinephrine, which effectively increased cardiac output despite a modest rise in pulmonary artery pressure. Milrinone, a phosphodiesterase‐3 inhibitor, was used for its dual role as an inotrope and pulmonary vasodilator. It consistently improved RV function and reduced PVR, although systemic hypotension was a frequent side effect that required concurrent vasopressor support with agents such as NE or AVP.

### 4.4. Pulmonary Vasodilator Therapy and Afterload Reduction

Reducing RV afterload was a key therapeutic goal, achieved by targeting elevated PVR, ensuring adequate oxygenation, correcting hypercapnia and acidosis, and minimizing airway pressures [11]. Pulmonary vasodilator therapy played a central role in this strategy. These agents act through NO, prostacyclin, or endothelin pathways to counteract hypoxic vasoconstriction and support RV function. Pulmonary vasodilators were administered only after stabilizing RV output to avoid systemic hypotension and the risk of pulmonary edema, especially in patients with elevated pulmonary venous pressures. Inhaled NO was used for its selective, short‐acting pulmonary vasodilatory effect. It improved oxygenation, reduced PVR, and decreased vasopressor requirements without significant systemic hypotension [11]. For synergistic benefit, inhaled epoprostenol (a prostacyclin analog) was combined with NO, resulting in enhanced pulmonary vasodilation, reducing PVR, and further unloading of the RV. We favored the use of nebulized rather than systemic prostanoids, as systemic administration may exacerbate hypoxemia and induce systemic hypotension, risks that are particularly concerning in hemodynamically unstable patients.

### 4.5. Mechanical Ventilation Strategies in RV Dysfunction

Mechanical ventilation has a significant impact on RV function, and inappropriate ventilator settings can worsen hemodynamics. Positive pressure ventilation, particularly with elevated PEEP or plateau pressures, increases intrathoracic pressure, reduces venous return, and raises RV afterload [12]. Both lung overdistension and collapse from high plateau pressures or atelectasis, respectively, can compromise pulmonary blood flow and exacerbate RV failure. Additionally, hypoxia and hypercapnia contribute to pulmonary vasoconstriction, further increasing PVR and RV strain, with acidosis amplifying these effects. In our patient, mechanical ventilation was initiated cautiously using lung protective strategies in accordance with the SAVIOR algorithm, including low tidal volumes (5 mL/kg ideal body weight), zero pressure support (0/0), and 100% FiO_2_ [13]. Settings were then gradually titrated to maintain SpO_2_ between 88% and 92%. This approach minimized dead space ventilation and avoided extremes in lung volume that could increase RV workload.

### 4.6. RV Function Assessment

In our case, we used TAPSE to assess interval changes in RV function following treatment. TAPSE is a widely accepted echocardiographic measure of RVSF that reflects longitudinal motion of the tricuspid annulus during systole. Its clinical appeal lies in its simplicity, reproducibility, and applicability even in critically ill patients. TAPSE is obtained from the apical four‐chamber view using M‐mode and is less dependent on image quality than other RV indices.

The patient′s initial echocardiogram revealed a moderately dilated RV (RV base diameter of 4.29 cm) with severely impaired systolic function and a TAPSE of 11.1 mm. A TAPSE value < 16 mm indicates RV systolic dysfunction and correlates well with radionuclide derived RV ejection fraction, 2D fractional area change, and other global RV performance metrics [14]. Even in normotensive patients, a reduced TAPSE has been linked to increased short‐term mortality and adverse outcomes. It is typically used in conjunction with other echocardiographic markers of RV strain, such as RV dilation and septal flattening, to assess severity and guide management.

In our case a repeat study performed 3 days posttreatment showed improved RV dimensions (RV base: 3.08 cm) and a TAPSE of 19.8 mm, consistent with significant recovery of RV function. Recovery of TAPSE after initial dysfunction is a favorable prognostic sign, indicating improved RV contractility and reduced afterload. Although TAPSE is angle dependent and reflects longitudinal RV motion rather than global RV function, its simplicity and reproducibility make it useful for serial bedside therapeutic response in critically ill patients. Conversely, persistently low TAPSE is associated with ongoing RV impairment and worse outcomes. Notably, TAPSE values > 20 mm carry a very low risk of 30‐day PE‐related mortality or need for rescue thrombolysis [15].

### 4.7. Neuroprotection After Cardiac Arrest

Despite prolonged resuscitation, the patient recovered without apparent neurologic deficit. This favorable outcome likely reflected high quality CPR, rapid restoration of circulation, correction of severe hypoxemia and shock, and adherence to postarrest supportive care principles. Postcardiac arrest brain injury is driven by global ischemia and reperfusion injury, including excitotoxicity, mitochondrial dysfunction, oxidative stress, and inflammation [16]. Current postarrest care emphasizes prevention of fever, avoidance of hypoxia and hypotension, maintenance of normocapnia, and avoidance of severe glucose derangements. In this case, aggressive cardiopulmonary stabilization and ICU level postarrest care likely contributed to neurologic recovery despite the prolonged duration of resuscitation [17–19].

## 5. Conclusion

Massive PE resulting in cardiac arrest portends a poor prognosis with a high mortality that far exceeds the mortality of ACS. This case illustrates that, in massive PE with cardiac arrest and persistent obstructive shock despite systemic thrombolysis, a sequential rescue strategy using repeat thrombolysis, attempted thrombectomy, and catheter‐directed thrombolysis may achieve survival with neurologic recovery, but with an uncertain bleeding risk without standardized dosing guidance. This outcome highlights the potential benefit of a timely, aggressive, and multidisciplinary approach in the management of massive PE with cardiac arrest.

## Funding

No funding was received for this manuscript.

## Disclosure

All authors have read and approved the final version of the manuscript. Jeffrey Valencia Uribe had full access to all of the data in this case and takes complete responsibility for the integrity of the case and the accuracy of the manuscript. The lead author affirms that this manuscript is an honest, accurate, and transparent account of the study being reported; that no important aspects of the case have been omitted; and that any discrepancies from the case as planned (and, if relevant, registered) have been explained.

## Consent

Written informed consent was obtained from the patient prior to the preparation of this manuscript.

## Conflicts of Interest

The authors declare no conflicts of interest.

## Data Availability

Data sharing is not applicable to this article as no datasets were generated or analyzed during the current study.

## References

[1] Kearon C. , Akl E. A. , Comerota A. J. , Prandoni P. , Bounameaux H. , Goldhaber S. Z. , Nelson M. E. , Wells P. S. , Gould M. K. , Dentali F. , Crowther M. , and Kahn S. R. , Antithrombotic Therapy for VTE Disease: Antithrombotic Therapy and Prevention of Thrombosis, 9th ed: American College of Chest Physicians Evidence-Based Clinical Practice Guidelines, Chest. (2012) 141, no. 2, e419S–e496S, 10.1378/chest.11-2301, 22315268. 22315268 PMC3278049

[2] Sharifi M. , Berger J. , Beeston P. , Bay C. , Vajo Z. , Javadpoor S. , and “PEAPETT” investigators , Pulseless Electrical Activity in Pulmonary Embolism Treated With Thrombolysis (From the "PEAPETT" Study), American Journal of Emergency Medicine. (2016) 34, no. 10, 1963–1967, 10.1016/j.ajem.2016.06.094, 27422214. 27422214

[3] Stevens S. M. , Woller S. C. , Baumann Kreuziger L. , Bounameaux H. , Doerschug K. , Geersing G. J. , Huisman M. V. , Kearon C. , King C. S. , Knighton A. J. , Lake E. , Murin S. , Vintch J. R. E. , Wells P. S. , and Moores L. K. , Executive Summary, Chest. (2021) 160, no. 6, 2247–2259, 10.1016/j.chest.2021.07.056. 34352279

[4] Panchal A. R. , Bartos J. A. , Cabañas J. G. , Donnino M. W. , Drennan I. R. , Hirsch K. G. , Kudenchuk P. J. , Kurz M. C. , Lavonas E. J. , Morley P. T. , O’Neil B. J. , Peberdy M. A. , Rittenberger J. C. , Rodriguez A. J. , Sawyer K. N. , Berg K. M. , Arafeh J. , Benoit J. L. , Chase M. , Fernandez A. , de Paiva E. F. , Fischberg B. L. , Flores G. E. , Fromm P. , Gazmuri R. , Gibson B. C. , Hoadley T. , Hsu C. H. , Issa M. , Kessler A. , Link M. S. , Magid D. J. , Marrill K. , Nicholson T. , Ornato J. P. , Pacheco G. , Parr M. , Pawar R. , Jaxton J. , Perman S. M. , Pribble J. , Robinett D. , Rolston D. , Sasson C. , Satyapriya S. V. , Sharkey T. , Soar J. , Torman D. , von Schweinitz B. , Uzendu A. , Zelop C. M. , and Magid D. J. , Part 3: Adult Basic and Advanced Life Support: 2020 American Heart Association Guidelines for Cardiopulmonary Resuscitation and Emergency Cardiovascular Care, Circulation. (2020) 142, no. 16_supplement_2, S366–S468, 10.1161/CIR.0000000000000916, 33081529. 33081529

[5] Giri J. , Sista A. K. , Weinberg I. , Kearon C. , Kumbhani D. J. , Desai N. D. , Piazza G. , Gladwin M. T. , Chatterjee S. , Kobayashi T. , Kabrhel C. , Barnes G. D. , On behalf of the American Heart Association Council on Peripheral Vascular Disease; Council on Arteriosclerosis , Thrombosis and Vascular Biology; Council on Cardiopulmonary, Critical Care , and Perioperative and Resuscitation; and Council on Cardiovascular Surgery and Anesthesia , Interventional Therapies for Acute Pulmonary Embolism: Current Status and Principles for the Development of Novel Evidence: A Scientific Statement From the American Heart Association, Circulation. (2019) 140, no. 20, e774–e801, 10.1161/CIR.0000000000000707, 31585051. 31585051

[6] Piazza G. , Hohlfelder B. , Jaff M. R. , Ouriel K. , Engelhardt T. C. , Sterling K. M. , Jones N. J. , Gurley J. C. , Bhatheja R. , Kennedy R. J. , Goswami N. , Natarajan K. , Rundback J. , Sadiq I. R. , Liu S. K. , Bhalla N. , Raja M. L. , Weinstock B. S. , Cynamon J. , Elmasri F. F. , Garcia M. J. , Kumar M. , Ayerdi J. , Soukas P. , Kuo W. , Liu P. Y. , Goldhaber S. Z. , and SEATTLE II Investigators , A Prospective, Single-Arm, Multicenter Trial of Ultrasound-Facilitated, Catheter-Directed, Low-Dose Fibrinolysis for Acute Massive and Submassive Pulmonary Embolism: The SEATTLE II Study, JACC Cardiovascular Interventions. (2015) 8, no. 10, 1382–1392, 10.1016/j.jcin.2015.04.020, 26315743. 26315743

[7] Sag S. , Nas O. F. , Kaderli A. A. , Ozdemir B. , Baran İ. , Erdoğan C. , Gullulu S. , Hakyemez B. , and Aydinlar A. , Catheter-Directed Ultrasound-Accelerated Thrombolysis May Be Life-Saving in Patients With Massive Pulmonary Embolism After Failed Systemic Thrombolysis, Journal of Thrombosis and Thrombolysis. (2016) 42, no. 3, 322–328, 10.1007/s11239-016-1370-3, 27129723. 27129723

[8] Ozcinar E. , Cakici M. , Dikmen Yaman N. , Baran C. , Aliyev A. , Inan B. , Durdu S. , Akar A. R. , and Sirlak M. , Thrombus Resolution and Right Ventricular Functional Recovery Using Ultrasound-Accelerated Thrombolysis in Acute Massive and Submassive Pulmonary Embolism, International Angiology: A Journal of the International Union of Angiology. (2017) 36, no. 5, 428–437, 10.23736/S0392-9590.17.03775-0, 28206731. 28206731

[9] Dumantepe M. , Uyar I. , Teymen B. , Ugur O. , and Enc Y. , Improvements in Pulmonary Artery Pressure and Right Ventricular Function After Ultrasound-Accelerated Catheter-Directed Thrombolysis for the Treatment of Pulmonary Embolism, Journal of Cardiac Surgery: Including Mechanical and Biological Support for the Heart and Lungs. (2014) 29, no. 4, 455–463, 10.1111/jocs.12354, 24827636. 24827636

[10] Mahboob H. B. and Denney B. W. , Double Bolus Alteplase Therapy During Cardiopulmonary Resuscitation for Cardiac Arrest due to Massive Pulmonary Embolism Guided by Focused Bedside Echocardiography, Case Reports in Critical Care. (2018) 2018, 7986087, 10.1155/2018/7986087, 29755795. 29755795 PMC5884296

[11] Price L. C. , Wort S. J. , Finney S. J. , Marino P. S. , and Brett S. J. , Pulmonary Vascular and Right Ventricular Dysfunction in Adult Critical Care: Current and Emerging Options for Management: A Systematic Literature Review, Critical Care. (2010) 14, no. 5, 10.1186/cc9264, 20858239. PMC3219266 20858239

[12] McGuire W. C. , Sullivan L. , Odish M. F. , Desai B. , Morris T. A. , and Fernandes T. M. , Management Strategies for Acute Pulmonary Embolism in the ICU, Chest. (2024) 166, no. 6, 1532–1545, 10.1016/j.chest.2024.04.032, 38830402. 38830402

[13] Srour H. , Shy J. , Klinger Z. , Kolodziej A. , and Hatton K. W. , Airway Management and Positive Pressure Ventilation in Severe Right Ventricular Failure: SAVIOR Algorithm, Journal of Cardiothoracic and Vascular Anesthesia. (2020) 34, no. 1, 305–306, 10.1053/j.jvca.2019.05.046, 31257055. 31257055

[14] Rudski L. G. , Lai W. W. , Afilalo J. , Hua L. , Handschumacher M. D. , Chandrasekaran K. , Solomon S. D. , Louie E. K. , and Schiller N. B. , Guidelines for the Echocardiographic Assessment of the Right Heart in Adults: A Report From the American Society of Echocardiography, Journal of the American Society of Echocardiography. (2010) 23, no. 7, 685–713, 10.1016/j.echo.2010.05.010, 20620859. 20620859

[15] Pruszczyk P. , Goliszek S. , Lichodziejewska B. , Kostrubiec M. , Ciurzyński M. , Kurnicka K. , Dzikowska-Diduch O. , Palczewski P. , and Wyzgal A. , Prognostic Value of Echocardiography in Normotensive Patients With Acute Pulmonary Embolism, JACC: Cardiovascular Imaging. (2014) 7, no. 6, 553–560, 10.1016/j.jcmg.2013.11.004, 24412192. 24412192

[16] Katz A. , Broshanahan S. , Papadopoulos J. , Parnia S. , and Lam J. Q. , Pharmacologic Neuroprotection in Ischemic Brain Injury After Cardiac Arrest, Annals of the New York Academy of Sciences. (2022) 1507, no. 1, 49–59, 10.1111/nyas.14613. 34060087

[17] Geocadin R. G. , Wijdicks E. , Armstrong M. J. , Damian M. , Mayer S. A. , Ornato J. P. , Rabinstein A. , Suarez J. I. , Torbey M. T. , Dubinsky R. M. , and Lazarou J. , Practice Guideline Summary: Reducing Brain Injury Following Cardiopulmonary Resuscitation, Neurology. (2017) 88, no. 22, 2141–2149, 10.1212/WNL.0000000000003966, 28490655. 28490655 PMC5447399

[18] Bang H. J. , Youn C. S. , Park K. N. , Oh S. H. , Kim H. J. , Kim S. H. , and Park S. H. , Glucose Control and Outcomes in Diabetic and Nondiabetic Patients Treated With Targeted Temperature Management After Cardiac Arrest, PloS One. (2024) 19, no. 2, e0298632, 10.1371/journal.pone.0298632, 38330019. 38330019 PMC10852315

[19] Eastwood G. , Nichol A. D. , Hodgson C. , Parke R. L. , McGuinness S. , Nielsen N. , Bernard S. , Skrifvars M. B. , Stub D. , Taccone F. S. , Archer J. , Kutsogiannis D. , Dankiewicz J. , Lilja G. , Cronberg T. , Kirkegaard H. , Capellier G. , Landoni G. , Horn J. , Olasveengen T. , Arabi Y. , Chia Y. W. , Markota A. , Hænggi M. , Wise M. P. , Grejs A. M. , Christensen S. , Munk-Andersen H. , Granfeldt A. , Andersen G. Ø. , Qvigstad E. , Flaa A. , Thomas M. , Sweet K. , Bewley J. , Bäcklund M. , Tiainen M. , Iten M. , Levis A. , Peck L. , Walsham J. , Deane A. , Ghosh A. , Annoni F. , Chen Y. , Knight D. , Lesona E. , Tlayjeh H. , Svenšek F. , McGuigan P. J. , Cole J. , Pogson D. , Hilty M. P. , Düring J. P. , Bailey M. J. , Paul E. , Ady B. , Ainscough K. , Hunt A. , Monahan S. , Trapani T. , Fahey C. , and Bellomo R. , Mild Hypercapnia or Normocapnia After Out-of-Hospital Cardiac Arrest, New England Journal of Medicine. (2023) 389, no. 1, 45–57, 10.1056/NEJMoa2214552. 37318140

